# College Members whose deaths were reported at Council meetings between October 2016 and October 2018 – ERRATUM

**DOI:** 10.1192/bjb.2019.15

**Published:** 2019-04

We included in error Dr Thomas Mary McBride of Carrigawley, Letterkenny, Ireland in the *In Memoriam* list published in the February 2019 issue. The information supplied to us was inaccurate but records have now been updated. We apologise to Dr McBride for this unfortunate error.
